# Characteristics of influenza H13N8 subtype virus firstly isolated from Qinghai Lake Region, China

**DOI:** 10.1186/s12985-017-0842-1

**Published:** 2017-09-18

**Authors:** Jie Dong, Hong Bo, Ye Zhang, Libo Dong, Shumei Zou, Weijuan Huang, Jia Liu, Dayan Wang, Yuelong Shu

**Affiliations:** National Institute for Viral Disease Control and Prevention, China Center for Disease Control and Prevention, Key Laboratory for Medical Virology, National Health and Family Planning Commission, 155 Changbai Road, Changping District, Beijing, 102206 China

**Keywords:** Avian influenza virus, H13N8 subtype, Qinghai Lake, Reassortant

## Abstract

**Background:**

Since the highly pathogenic H5N1 influenza caused thousands of deaths of wild bird in this area in 2005, Qinghai Lake in China has become a hot spot for study of the influence of avian influenza to migratory wild birds. However, the ecology and evolution of low pathogenic avian influenza virus in this region are limited. This project-based avian influenza surveillance in Qinghai lake region was initiated in year 2012.

**Method:**

Samples of wild bird feces and lake surface water were collected in Qinghai Lake in year 2012.Virus isolation was conducted on embryonated chicken eggs. The influenza A virus was determined by rRT-PCR. Virus sequences were acquired by deep sequencing. The phylogenetic correlation and molecular characteristics of the viruses were analyzed. The virus growth and infection features, receptor binding preference were studied, and pathogenicity in vitro as well as.

**Results:**

Two H13N8 subtype influenza viruses were isolated. The viruses are phylogenetically belong to Eurasian lineage. Most of the genes are associated with gull origin influenza virus except PB1 gene, which is most probably derived from Anseriformes virus. The evidence of interspecies reassortment was presented. The two viruses have limited growth capacity on MDCK and A549 cells while grow well in embryonated eggs. The dual receptor binding features of the two viruses was shown up. The low pathogenic features were determined by trypsin dependence plaque formation assay.

**Conclusions:**

The two H13N8 subtype influenza viruses are highly associated with gull origin. The interspecies reassortment of H13 subtype virus among Anseriforme sand Charadriiformes wild birds emphasizes the importance of strengthening avian influenza surveillance in this region. This study is helpful to understand the ecology, evolution and transmission pattern of H13 subtype influenza virus globally.

**Electronic supplementary material:**

The online version of this article (10.1186/s12985-017-0842-1) contains supplementary material, which is available to authorized users.

## Background

Wild water birds usually can be classified as Anseriformes and Charadriiformes, which are represented by duck and gull respectively. They are the natural reservoirs for all avian influenza subtypes. Influenza A virus H13 subtype seems to be highly gull associated [1] and it is rare to isolate H13 viruses from Anseriformes, such as duck and goose. Since its isolation firstly reported in 1977 [2], the H13 subtype influenza has been divided into Eurasian and North American lineages according to the evolutional relationship [3]. The intercontinental and interspecies reassortment of viruses had happened occasionally [4, 5].

Wild aquatic birds serve as natural reservoirs harboring 16 Hemagglutinin (HA) and 9 Neuraminidase (NA) subtypes of influenza A virus. Influenza A viruses, except for highly pathogenic avian influenza H5 and H7 subtype viruses, usually cause mild or even asymptomatic infection among birds (known as low pathogenic avian influenza). The viruses in migratory wildfowl may spillover to birds or mammals, or reassort with other influenza A viruses, causing diseases with pandemic potential.

An outbreak caused by highly pathogenic avian influenza H5N1 resulted in more than 10,000 deaths of migratory birds in Qinghai Lake in year 2005 [6, 7]. The virus was then spread to Mongolia, Russian, Europe, and Africa along the migratory flyways in the following years of 2005–09 [8, 9]. This event is a typical example of the global transmission of avian influenza viruses. Since then, Qinghai Lake has become an animal and human public health concern due to its geographic location, which is major breeding site for migratory birds flying to Australia, India, Siberia and Southeast Asia via the Central Asian-Indian flyway and the East Asian-Australian flyway.

The data of the ecology and distribution of influenza A virus subtype, especially the low pathogenic avian influenza subtypes in the Qinghai Lake region is lacking. In view of this, the project-based avian influenza surveillance of relevant environmental samples in Qinghai Lake was implemented in year 2012 Samples of bird feces and lake surface water were collected in core areas of Qinghai Lake, such as Bird Island and Xiannvwan. A few of influenza A subtype viruses were isolated from collected samples, among which two H13N8 subtype influenza viruses were identified and their characteristics were studied.

## Methods

### Viruses

Fecal samples were collected and processed according to WHO manual [10]. The viruses were isolated in SPF(specific pathogen free) embryonated chicken eggs at 37°Cfor 3 days. The hemagglutination assay with 1% turkey erythrocytes in a PBS solution was used to test the viruses [10]. The viruses were confirmed by rRT-PCR based on type specific influenza M gene with Stratagene Mx3005P thermocycler using amplification protocol as steps of 45°Cfor 10 min and 95°Cfor 10 min and then 40 cycles of 95°Cfor 15 s and 60°Cfor 45 s. The viruses were purified on eggs by passage with limited dilution and the viruses were stored as stock viruses. The sequences of primers and probe are as (Forward prime:5’GACCRATCCTGTCACCTCTGAC3’, Reverse prime: 5’AGGGCATTYTGGACAAAKCGTCTA3’,Probe: ‘FAM-TGCAGTCCTCGCTCACTGGGCACG-BHQ1–3′).

### RNA extraction and genome sequencing

The viral RNA was extracted by An RNeasy Kit (Qiagen, Chartsworth, CA, USA). Double stranded DNA was synthesized based on a reverse transcription reaction using SuperScrip^th^III One-Step RT-PCR System(Invitrogen USA), The amplification steps of 45°Cfor 60 min and 94°Cfor 2 min and then 5 cycles of 94°Cfor 30s, 44°Cfor 30s and 68°Cfor 3 min and then 31 cycles of 94°Cfor 30s, 57°Cfor 30s and 68°Cfor 3 min, finally 68°Cfor 7 min.The primers are Uni12/inf-1 5′-GGGGGGAGCAAAAGCAGG-3′, Uni13/inf-1 5′-CGGGTTATTAGTAGAACAAGG-3′ The genome deep sequencing was done using Illumina system Nextera XT Library Prep Kit to fragment DNA and add adapters onto the DNA template. The detail protocol was followed as reference [11].

### Phylogenetic analysis

The MEGA7 (http://www.megasoftware.net/) was used to take multiple sequence alignment and phylogenetic analysis. The neighbor-joining method with 1000 bootstrap value was chosen as value for each gene phylogenetic analysis.

### Virus titration on different cell lines

Virus stocks were titrated on Human–type IIalveolar epithelial (A549), Madin-Darby canine kidney (MDCK), Porcine Kidney (PK15),embryonated chicken eggs respectively. The detailed protocols of virus titration referred to the WHO manual [10], TCID_50_(50% Tissue culture infective dose) and EID_50_(50% Egg infective dose) calculation were determined by using the Reed-Muench formula.

### Receptor binding analysis based on hemagglutination

Two types of blood cell were chosen to conduct the hemagglutination assay. Including 1% Turkey red blood cell (TRBC) withα2,3 andα2,6 sialic linked receptors, and 1%α2,3 specific sialidase treated TRBC which only containedα2,6 receptor. The properties of receptor binding were distinguished by virus hemagglutination difference. The treatment detail of blood cells was taken as reference [12]. Original 10% TRBC suspension in phosphate buffer solution (PBS) was treated by 625mUα2,3 specific sialidase (Takara Dalian, China) at 37°Cfor 30 min. Complete elimination ofα-2,3-receptor of treated TRBCs was confirmed by receptor staining and flow cytometry.

### Trypsin dependence assay

The viral plaque characteristics were determined with MDCK cells. MDCK cells were grown on 96-well culture plate with 3 × 10^4^ /well at 37 °C for 1 day. Serial dilutions of virus were inoculated on MDCK cells. 2–3 h later after virus absorption, the overlap medium(2 × DMEM and avicell) was placed. The overlap medium with or without TPCK treated trypsin (with final concentration of 2μg/ml) was prepared and placed respectively. After 2 days of inoculation, the cell plates were fixed by 4% paraformaldehyde in PBS solution at 4 °C 30mins. Then the cells were permeabilized. ELISA was performed with mouse monoclonal antibody against influenza type A (CDC –WHO kit used at 1:2000 in ELISA Buffer) as first antibody, and goat anti-mouse IgG (H + L) HRP conjugate (Biorad 172–1011 used at 1:1000 in ELISA Buffer) as second antibody. True Blue™ peroxidase substrate (KPL 50–78-02), and 0.03% H_2_O_2_(1:1000 of 30% solution) were added to present the plaque formation [13].

## Results

### Virus information

During the project-based surveillance in Qinghai Lake in Year 2012, a total of 796 wild birds related environmental samples were collected with 0.88% influenza A virus positive rate.7 strains of three influenza subtypes were isolated from wild bird feces of Qinghai Lake. Among these, two H13N8 viruses named as A/Environment/Qinghai lake/013/2012(H13N8) and A/Environment/Qinghai lake/166/2012 (H13N8) were identified. Full genome sequences of the two isolated influenza viruses have been uploaded to the Global Initiative on Sharing Avian Influenza Database (GISAID) under accession numbers EP11036520-EP11036535.

### Phylogenetic tree and homology analysis

The two H13N8 subtype avian influenza sequences were compared with related sequences in GenBank Database. The two H13N8 viruses showed 98–100% homology in all 8 segments. The most closely strains were identified based on nucleotide level (Table 1). 6/8 segments seem from gull origin except PB1 and HA gene segments. But the HA segment is derived from A/mallard/Korea/SH38–45/2010(H13N2) which was reported as reassortant virus with gull origin HA,M segments and the rest from wild duck.

**Table 1:** Nucleotide identity of two H13N8 viruses with the most closely related strains in GenBank Database

Gene segment	Strains in GenBank with high similarity	Nucleotide identity (%)	Accession number
HA	A/mallard/Korea/SH38–45/2010 (H13N8)	97.7	JX030406.1
NA	A/black-headed gull/Republic of Georgia/1/2011(H13N8)	98.7	CY185515.1
M	A/black-headed gull/Netherlands/8/2010 (H13N2)	99	KX977972.1
NS	A/yellow-legged gull/Republic of Georgia/1/2010(N2)	99.5	CY185313.1
NP	A/black-headed gull/Netherlands/10/2013 (H13N8)	98.7	KX978516.1
PA	A/yellow-legged gull/Republic of Georgia/1/2013(H13N8)	98.8	CY185630.1
PB1	A/wild bird/Wuhan/CDHN09/2015 (H6N2)	99	KU143516.1
PB2	A/yellow-legged gull/Republic of Georgia/1/2013(H13N8)	98.6	CY185632.1

**Fig. 1:**
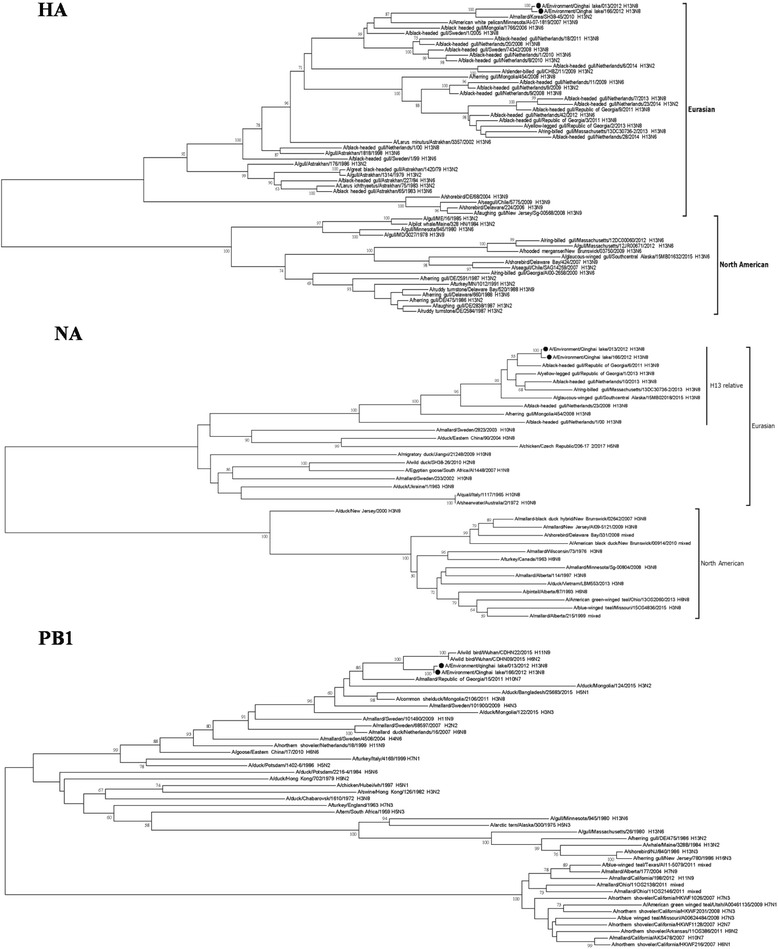
Phylogenetic trees of HA, NA and PB1 of two H13N8 viruses, .**a**) H13 gene **b**) N8 gene **c**) PB1 gene. Three segment full sequences were used to conduct phylogenetic tree using MEGA 7 with 1000 neighbor-joining replicates. Two H13N8 viruses isolated in Qinghai lake region are indicated by filled circles

Phylogenetic analysis of HA gene showed that there were two separate lineages, namely Eurasian and North American existed. The two H13N8 viruses belonged to the Eurasian lineage. N8 of two viruses were clustered in H13 relative strains of Eurasian lineage, and were comparatively far from N8 of other subtypes (Fig. 1). It was clear that, according to the evolution relationship, 5 of six internal genes presented PB2, NS, NP, PA and M were closely related to gull-originated viruses. Especially, PB2, NS and NP showed H13 and H16 specific features, which means that these genes were clustered together with H13 and H16 subtype viruses (Additional file 1). Interestingly, the PB1 gene evolution of two H13N8 viruses was quite complete. It is most probably derived from Anseriformes such as wild ducks, and far away from gull associated viruses (Fig. 1).

### Molecular characterization

The HA cleavage site sequences of the two H13N8 viruses were PAISNR↓GLF, which presented low pathogenic avian influenza properties. Q226L mutation of HA, which is related to human receptor binding preference, was not identified in two H13N8 viruses, but the S228, which showed human receptor binding preference, was demonstrated in HA protein of two H13N8 viruses. V135 and S136 substitutions of HA protein 130 loop might impact the receptor binding specificity, which is quite different from the firstly isolated H13N6 virus named as A/gull/Maryland/704/1977. The two H13N8 viruses have not shown mammalian adaptation mutations of PB2, such as E627K, D701N substitution, which indicated these viruses’ avian origin. The N30D, T215A substitutions of M1 protein were found in two H13N8 viruses, which is associated with increased pathogenicity of H5N1 virus in mice. There were no adamantine nor neuraminidase inhibitor resistance mutation found in M2 and NA proteins of the two H13N8 viruses (Table 2), indicating these two types of antiviral drug are still sensitive to two H13N8 viruses.

**Table 2:** Specific sites analysis of two H13N8 virus proteins

Protein	Mutation sites (aa)	Viruses		Possible function
		A/Environment/Qinghai Lake/013/2012	A/Environment/Qinghai Lake/166/2012	
HA	T135 V	V	V	Receptor binding specificity relative 130 bp
	T136S	S	S	
	V186 N	V	V	Human receptor binding preference of H13 subtype
	E190D	E	E	Human receptor binding preference
	Q226L	Q	Q	Human receptor binding shift of H2,3,5 subtypes
	G228S	S	S	
		PAISNR↓GLF	PAISNR↓GLF	HA cleavage site
PB2	E627K	E	E	Mammalian adaption mutations
	D701N	D	D	
M1	N30D	D	D	Increase pathogenicity of H5N1 to mice
	T215A	A	A	
M2	S31 N	S	S	Adamantine resistance mutation
NA	E119V	E	E	Neuraminidase inhibitor resistance mutation
	R292K	R	R	
	R152V	R	R	
	H274Y	H	H	

*specific sites related to virus pathogenicity, virulence which have been published by other studies

### Virus titration on different types of cells

To understand the virus growth characteristics, we titrated two stock viruses on different cells: A549, MDCK, PK15, and embryonated eggs (Table 3). The two H13N8 viruses presented same TCID_50_ on MDCK and A549 cells. The EID_50_ and TCID_50_ on PK15 of two H13N8 viruses presented a little difference. The two H13N8 viruses showed preferential replication on eggs that indicated avian influenza properties.

**Table 3:** H13N8 Virus titration on different types of cells

Culture condition	Virus titration	
	A/Environment/Qinghai Lake/013/2012 (H13N8)	A/Environment/Qinghai Lake/166/2012 (H13N8)
Embryonated eggs	10^8^EID_50_/200 μl	10^7.33^EID_50_/200 μl
MDCK	10^3.25^TCID_50_/100 μl	10^3.25^TCID_50_/100 μl
A549	10^2.75^TCID_50_/100 μl	10^2.75^TCID_50_/100 μl
PK15	10^5^TCID_50_/100 μl	10^4.5^TCID_50_/100 μl

### Receptor binding specificity

The two H13N8 viruses presented reduced HA titers(4 fold decrease) with α2,3 -sialidase treated turkey red blood cells, which supportedα2,6 linked sialylated glycans left and α2,3 linked sialylated glycans removed. Two human origin H1N1 and H9N2 virus strains have same HA titers with α2,3 -sialidase treated or not treated turkey blood cells. The HA titer of the H5N1 strain have significant difference(from 1:128 to negative) when binding with α2,3 -sialidase treated or not treated turkey blood cells, which means it only hasα2,3 avian influenza receptor binding property, although it was isolated from human being. Our results indicated that these two H13N8 viruses have dual receptor binding property, which was also presented by A/Anhui/1/2013 RG (H7N9) (Table 4). Dual receptor binding feature of H7N9 viruses such as A/Anhui/1/2013 has been confirmed by a study described as reference [14].

**Table 4:** Receptor specificity of H13 viruses by hemagglutination assay

Viruses	HA titers (1:X)		Binding^a^	
	TRBC	TRBC treated with sialidase	α2,3-SA	α2,6-SA
A/Environment/Qinghai Lake/013/2012 (H13N8)	64	16	+	+
A/Environment/Qinghai Lake/166/2012 (H13N8)	256	64	+	+
A/Anhui/1/2005 RG (H5N1)	128	–	+	–
A/Hunan/44558/2014 (H9N2)	>2048	>2048	–	+
A/Brisbane/59/2007 (H1N1)	64	64	–	+
A/Anhui/1/2013 RG (H7N9)	128	32	+	+

a binding is shown as “+”, and no binding is shown as “-”

### Trypsin dependence properties

**Fig. 2:**
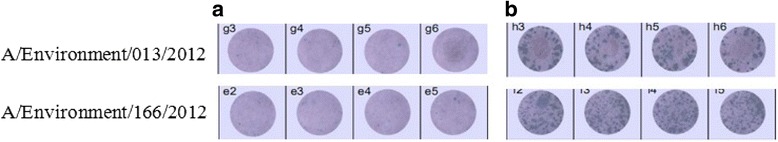
Trypsin dependence plague formation assay of two H13N8 viruses

Trypsin independence of virus culture is one of the properties of highly pathogenic avian influenza virus due to its multi-basic cleavage motif of HA that can be cleaved by furin-like proteases, which is persisted in all vertebrate cells. However, the culture of low pathogenic avian influenza viruses must add the exogenous trypsin in the viral growing media. We determined the two H13N8 virus trypsin dependence properties by plague formation assay. The plaque was presented with trypsin and there was no clear plaque formation in the absence of trypsin that indicated two viruses were low pathogenic to avian (Fig. 2). Four parallel wells named g3-g6 and 4 parallel wells named h3–6 indicated A/Environment/013/2012 without trypsin and with trypsin respectively. The e2–5 and f2–5 were represented the virus A/Environment/166/2012 without and with trypsin.

## Discussion

Influenza A virus surveillance among gulls was systematically conducted in Netherlands, Norway and Georgia [15, 17, 18]. The annual epidemics in gulls caused by H13 and H16 subtype viruses often occurred. First year gull is more susceptive of H13 and H16 infection than gulls older than one year [16]. Adult gulls had antibody reaction against H13 and H16 viruses and H16 antibodies were most common [17]. To date, gulls are considered the natural reservoir of influenza A H13 and H16 subtype viruses and gulls also host other subtypes of influenza A virus with diversity. It is possible to cause potential reassortment within species. Gulls sharing same habitats with wild ducks and shorebirds will increase the risk of cross-species transmission or reassortment of viruses resulting in novel subtype viruses.

Usually, based on its geographic location, influenza H13 subtype viruses are separated into Eurasian and North American lineages. Substantial genetic reassortment of the two continents had happened through wild bird migration. The overlapping places of wild bird flyways became the hot spot for avian influenza ecological and epidemiological study. The surveillance took place in Georgia showed Georgian influenza A virus subtype distribution was different by wild bird flyways. In East Africa and West Asia, H7, 11, 13 and N6 were more concentrated [18]. Most genes of our two H13N8 viruses are closely related to Georgian isolates that support the concept of virus transmission by wild bird through long-distance migration of east Asia and west Asia flyway. Qinghai lake is located in the crossing places of three wild bird migratory flyways: central Asia, east Asia-Australia, east Africa-west Asia. Hence its importance of avian influenza ecology, evolution should be paid more attention.

Although influenza H13 subtype virus was mostly associated with gulls, the finding of interspecies reassortment with genes from Anseriformes (such as mallard) viruses have also been reported [19]. In our study, PB1 genes of the two H13N8 viruses were phylogenetically relevant to the viruses from Anseriformes, which is deemed an evidence of interspecies reassortment.

H13 subtype influenza virus can infect gulls to induce antibody reaction [20]. The susceptibility to H13 virus is presented differently among avian species. Gulls are highly susceptible, ducks and turkeys are resistant to some strains, and chickens are refractory to infections of all strains [21]. The tissue tropism and pathology of H13 natural infection of black-headed gulls showed that H13 virus has adapted to gulls with minimal pathogenicity, with non-clinical signs [22]. In addition, gull-related H13 subtype influenza viruses also caused the infection and stranding of marine mammals such as whales [23].

In our study, the two H13N8 showed dual receptor binding properties, which means that they have a capacity to attach to both human receptor and avian receptor. These viruses may infect human being under certain suitable conditions as binding a 2, 6 linked sialic acids (SA) is a pre- requirement for AIV transmission to humans. The molecular basic of receptor binding specificity is subtyped dependent. Different subtypes, or even different strains of same type, might have different molecular markers of receptor binding specificity. The substitution or mutation of HA protein relative to receptor binding preference was concentrated on sites 226,228,186,190 and 135–137. The soluble H13 HA glycoprotein of A/gull/Maryland/704/1977(H13N6) was purified, and its receptor binding specificity was characterized as binding exclusively of the avian a 2, 3 linked sialic acids receptor [24], which was dissimilar with the two H13N8 viruses. This is probably due to the different amino acids composition of position 135–136 of HA. We should monitor the ecology of this type of virus in birds and potential reassortment with other subtypes of avian influenza.

According to the criteria of pathogenicity of influenza A virus adopted by OIE, the highly pathogenic influenza was determined by the intravenous pathogenicity index (IVPI) test on chickens. In terms of deduction of in-vivo tests, the determination of the cleavage site of HA by sequencing and trypsin dependence assay should be initiatively taken [25]. We found that the cleavage site of HA of the two H13N8 viruses was mono-basic cleavage sites, which contained only one basic amino acid in the critical position PAISN**R**↓GLF. And the virus growth presented clearly trypsin dependence due to not having multi-basic amino acids at the cleavage site of HA. The low pathogenicity of the two H13N8 viruses was confirmed by the virus inoculation on eggs. No egg death was shown.

Virus titration on different cell types can reflect virus growth and infection abilities. The two H13N8 viruses presented low TCID_50_ value on A549 and MDCK(10^3.25/100ul, 10^2.75/100ul)with limited growth characteristics. The reason of poor virus replication on A549 and MDCK is probably that these cell lines are of mammalian origin. These viruses can reach higher titer on embryonated eggs that showed avian tissues preference. This result will be ideal basis for avian influenza H13 subtype virus isolation and propagation.

To our knowledge, this is the first time that H13N8 subtype influenza virus isolated from Qinghai lake region is reported. Although there is no evidence showing that this low pathogenic avian influenza subtype virus is a risk to wild birds or human beings, it is helpful to understand the ecology and evolution of avian influenza virus in this region, and to provide foundation for the study of correlation between wild bird migration and virus transmission.

## Conclusions

In conclusion, two H13N8 subtype influenza viruses were firstly isolated from Qinghai Lake region in China. Its phylogenetic relationship indicated that they are highly associated with gull origin except PB1 gene which is most likely derived from Anseriformes birds. The interspecies reassortment was presented. Low pathogenicity, limited grown capacity on mammalian cells of these viruses showed that H13N8 subtype virus is low risk virus to both animals and human beings. This study is a clue to better understand the ecology, evolution and transmission pattern of H13 subtype influenza virus globally.

## Additional file

Additional file 1: Phylogenetic trees of a (PB2), b (PA), c (NP), d (M), and e (NS) of two H13N8 viruses. Full sequences were used to conduct phylogenetic tree using MEGA 7 with 1000 neighbor-joining replicates. Two H13N8 viruses isolated in Qinghai lake region are indicated by filled circles. (PDF 1 MB)

## Abbreviations

A549: Human–type IIalveolar epithelial
EID_50_: 50% Egg infective dose
HA: Hemagglutinin
MDCK: Madin-Darby canine kidney
NA: Neuraminidase
PK15: Porcine Kidney
SPF: Specific pathogen free
TCID_50_: 50% Tissue culture infective dose
TRBC: Turkey red blood cell
