# Supplementary material for: Characteristics of influenza H13N8 subtype virus firstly isolated from Qinghai Lake Region, China
Source: Virol J. 2017 Sep 18;14:180. doi: 10.1186/s12985-017-0842-1 (PMC5604506; doi:10.1186/s12985-017-0842-1)
Supplement: Additional file 1: — Phylogenetic trees of a (PB2), b (PA), c (NP), d (M), and e (NS) of two H13N8 viruses. Full sequences were used to conduct phylogenetic tree using MEGA 7 with 1000 neighbor-joining replicates. Two H13N8 viruses isolated in Qinghai lake region are indicated by filled circles. (PDF 1 MB) [file 12985_2017_842_MOESM1_ESM.pdf]

a

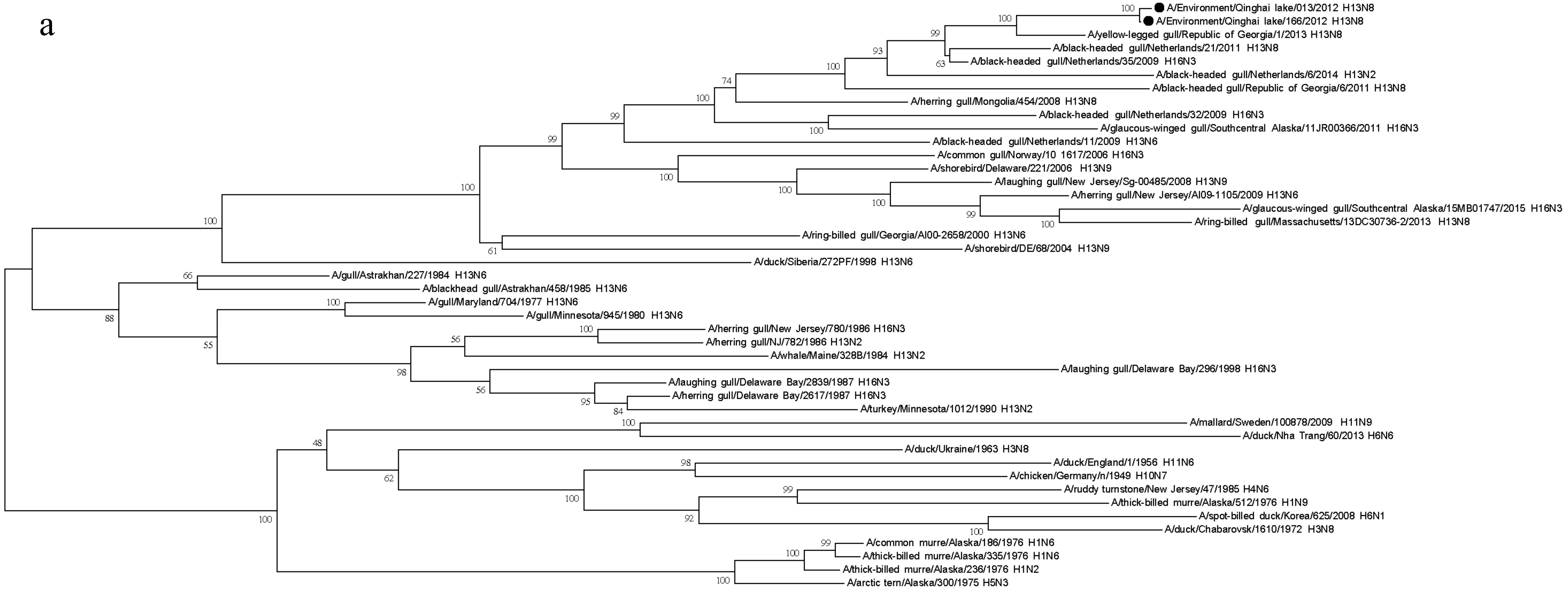
A/Environment/Qinghai lake/013/2012 H13N8
A/Environment/Qinghai lake/166/2012 H13N8
A/yellow-legged gull/Republic of Georgia/1/2013 H13N8
A/black-headed gull/Netherlands/21/2011 H13N8
A/black-headed gull/Netherlands/35/2009 H16N3
A/black-headed gull/Netherlands/6/2014 H13N2
A/black-headed gull/Republic of Georgia/6/2011 H13N8
A/herring gull/Mongolia/454/2008 H13N8
A/black-headed gull/Netherlands/32/2009 H16N3
A/glaucous-winged gull/Southcentral Alaska/11JR00366/2011 H16N3
A/black-headed gull/Netherlands/11/2009 H13N6
A/common gull/Norway/10 1617/2006 H16N3
A/shorebird/Delaware/221/2006 H13N9
A/laughing gull/New Jersey/Sg-00485/2008 H13N9
A/herring gull/New Jersey/AI09-1105/2009 H13N6
A/glaucous-winged gull/Southcentral Alaska/15MB01747/2015 H16N3
A/ring-billed gull/Massachusetts/13DC30736-2/2013 H13N8
A/ring-billed gull/Georgia/AI00-2658/2000 H13N6
A/shorebird/DE/68/2004 H13N9
A/duck/Siberia/272PF/1998 H13N6
A/gull/Astrakhan/227/1984 H13N6
A/blackhead gull/Astrakhan/458/1985 H13N6
A/gull/Maryland/704/1977 H13N6
A/gull/Minnesota/945/1980 H13N6
A/herring gull/New Jersey/780/1986 H16N3
A/herring gull/NJ/782/1986 H13N2
A/whale/Maine/328B/1984 H13N2
A/laughing gull/Delaware Bay/296/1998 H16N3
A/laughing gull/Delaware Bay/2839/1987 H16N3
A/herring gull/Delaware Bay/2617/1987 H16N3
A/turkey/Minnesota/1012/1990 H13N2
A/mallard/Sweden/100878/2009 H11N9
A/duck/Nha Trang/60/2013 H6N6
A/duck/Ukraine/1963 H3N8
A/duck/England/1/1956 H11N6
A/chicken/Germany/n/1949 H10N7
A/ruddy turnstone/New Jersey/47/1985 H4N6
A/thick-billed murre/Alaska/512/1976 H1N9
A/spot-billed duck/Korea/625/2008 H6N1
A/duck/Chabarovsk/1610/1972 H3N8
A/common murre/Alaska/186/1976 H1N6
A/thick-billed murre/Alaska/335/1976 H1N6
A/thick-billed murre/Alaska/236/1976 H1N2
A/arctic tern/Alaska/300/1975 H5N3

b

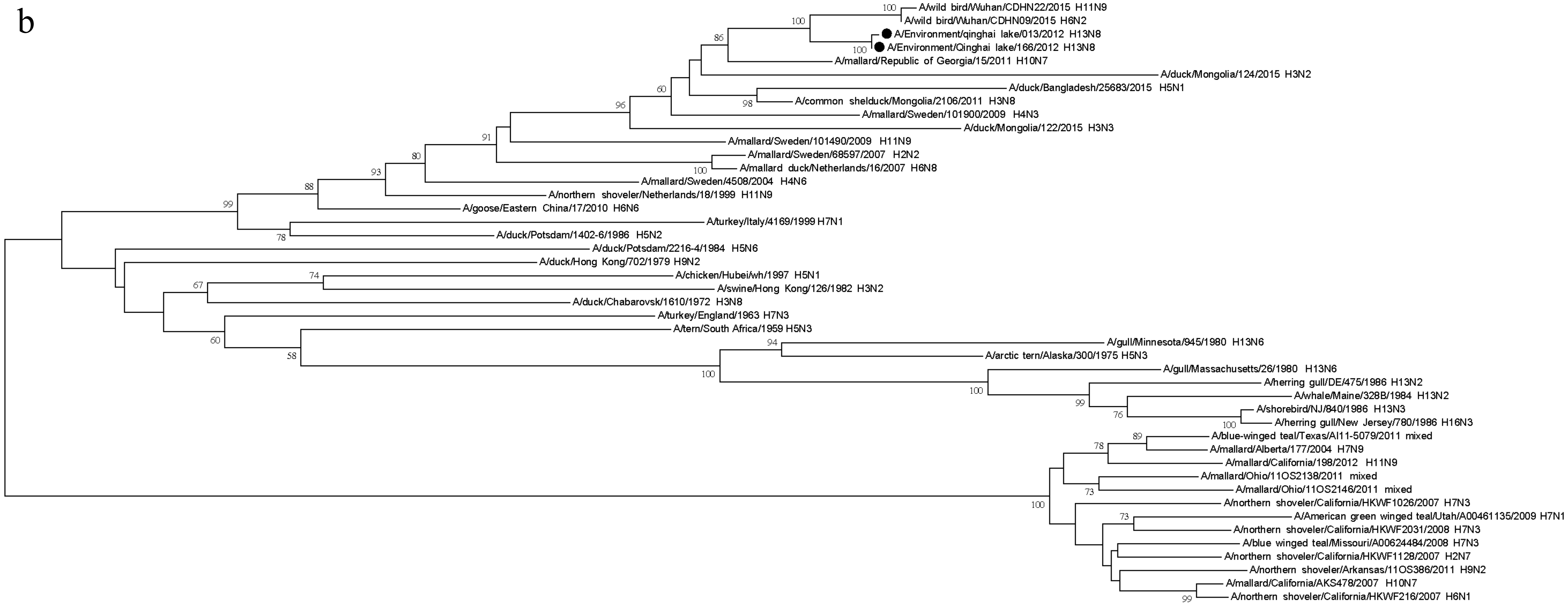
A/wild bird/Wuhan/CDHN22/2015 H11N9
A/wild bird/Wuhan/CDHN09/2015 H6N2
A/Environment/qinghai lake/013/2012 H13N8
A/Environment/Qinghai lake/166/2012 H13N8
A/mallard/Republic of Georgia/15/2011 H10N7
A/duck/Mongolia/124/2015 H3N2
A/duck/Bangladesh/25683/2015 H5N1
A/common shelduck/Mongolia/2106/2011 H3N8
A/mallard/Sweden/101900/2009 H4N3
A/duck/Mongolia/122/2015 H3N3
A/mallard/Sweden/101490/2009 H11N9
A/mallard/Sweden/68597/2007 H2N2
A/mallard duck/Netherlands/16/2007 H6N8
A/mallard/Sweden/4508/2004 H4N6
A/northern shoveler/Netherlands/18/1999 H11N9
A/goose/Eastern China/17/2010 H6N6
A/turkey/Italy/4169/1999 H7N1
A/duck/Potsdam/1402-6/1986 H5N2
A/duck/Potsdam/2216-4/1984 H5N6
A/duck/Hong Kong/702/1979 H9N2
A/chicken/Hubei/wh/1997 H5N1
A/swine/Hong Kong/126/1982 H3N2
A/duck/Chabarovsk/1610/1972 H3N8
A/turkey/England/1963 H7N3
A/tern/South Africa/1959 H5N3
A/gull/Minnesota/945/1980 H13N6
A/arctic tern/Alaska/300/1975 H5N3
A/gull/Massachusetts/26/1980 H13N6
A/herring gull/DE/475/1986 H13N2
A/whale/Maine/328B/1984 H13N2
A/shorebird/NJ/840/1986 H13N3
A/herring gull/New Jersey/780/1986 H16N3
A/blue-winged teal/Texas/AI11-5079/2011 mixed
A/mallard/Alberta/177/2004 H7N9
A/mallard/California/198/2012 H11N9
A/mallard/Ohio/11OS2138/2011 mixed
A/mallard/Ohio/11OS2146/2011 mixed
A/northern shoveler/California/HKWF1026/2007 H7N3
A/American green winged teal/Utah/A00461135/2009 H7N1
A/northern shoveler/California/HKWF2031/2008 H7N3
A/blue winged teal/Missouri/A00624484/2008 H7N3
A/northern shoveler/California/HKWF1128/2007 H2N7
A/northern shoveler/Arkansas/11OS386/2011 H9N2
A/mallard/California/AKS478/2007 H10N7
A/northern shoveler/California/HKWF216/2007 H6N1

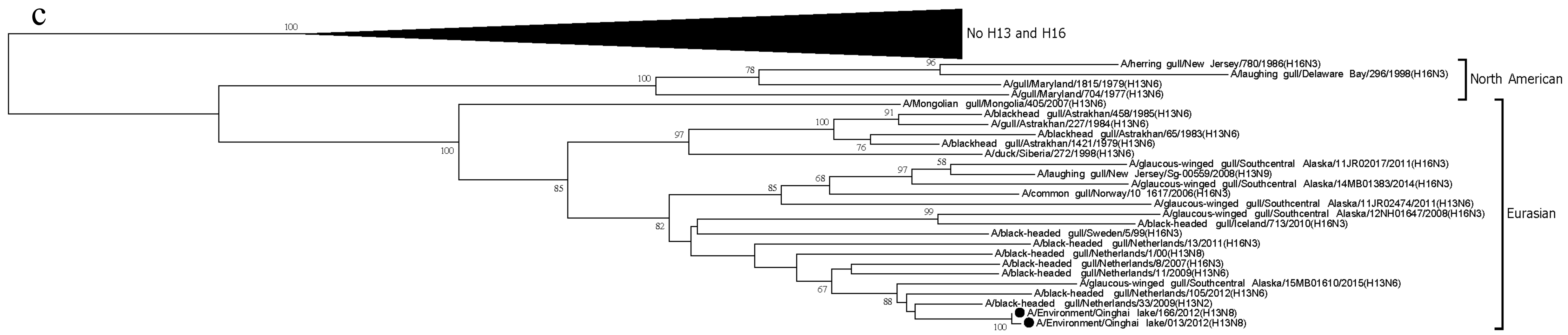

c
No H13 and H16
A/herring gull/New Jersey/780/1986(H16N3)
A/laughing gull/Delaware Bay/296/1998(H16N3)
A/gull/Maryland/1815/1979(H13N6)
A/gull/Maryland/704/1977(H13N6)
North American
A/Mongolian gull/Mongolia/405/2007(H13N6)
A/blackhead gull/Astrakhan/458/1985(H13N6)
A/gull/Astrakhan/227/1984(H13N6)
A/blackhead gull/Astrakhan/65/1983(H13N6)
A/blackhead gull/Astrakhan/1421/1979(H13N6)
A/duck/Siberia/272/1998(H13N6)
A/glaucous-winged gull/Southcentral Alaska/11JR02017/2011(H16N3)
A/laughing gull/New Jersey/Sg-00559/2008(H13N9)
A/glaucous-winged gull/Southcentral Alaska/14MB01383/2014(H16N3)
A/common gull/Norway/10 1617/2006(H16N3)
A/glaucous-winged gull/Southcentral Alaska/11JR02474/2011(H13N6)
A/glaucous-winged gull/Southcentral Alaska/12NH01647/2008(H16N3)
A/black-headed gull/Iceland/713/2010(H16N3)
A/black-headed gull/Sweden/5/99(H16N3)
A/black-headed gull/Netherlands/13/2011(H16N3)
A/black-headed gull/Netherlands/1/00(H13N8)
A/black-headed gull/Netherlands/8/2007(H16N3)
A/black-headed gull/Netherlands/11/2009(H13N6)
A/glaucous-winged gull/Southcentral Alaska/15MB01610/2015(H13N6)
A/black-headed gull/Netherlands/105/2012(H13N6)
A/black-headed gull/Netherlands/33/2009(H13N2)
A/Environment/Qinghai lake/166/2012(H13N8)
A/Environment/Qinghai lake/013/2012(H13N8)
Eurasian

d

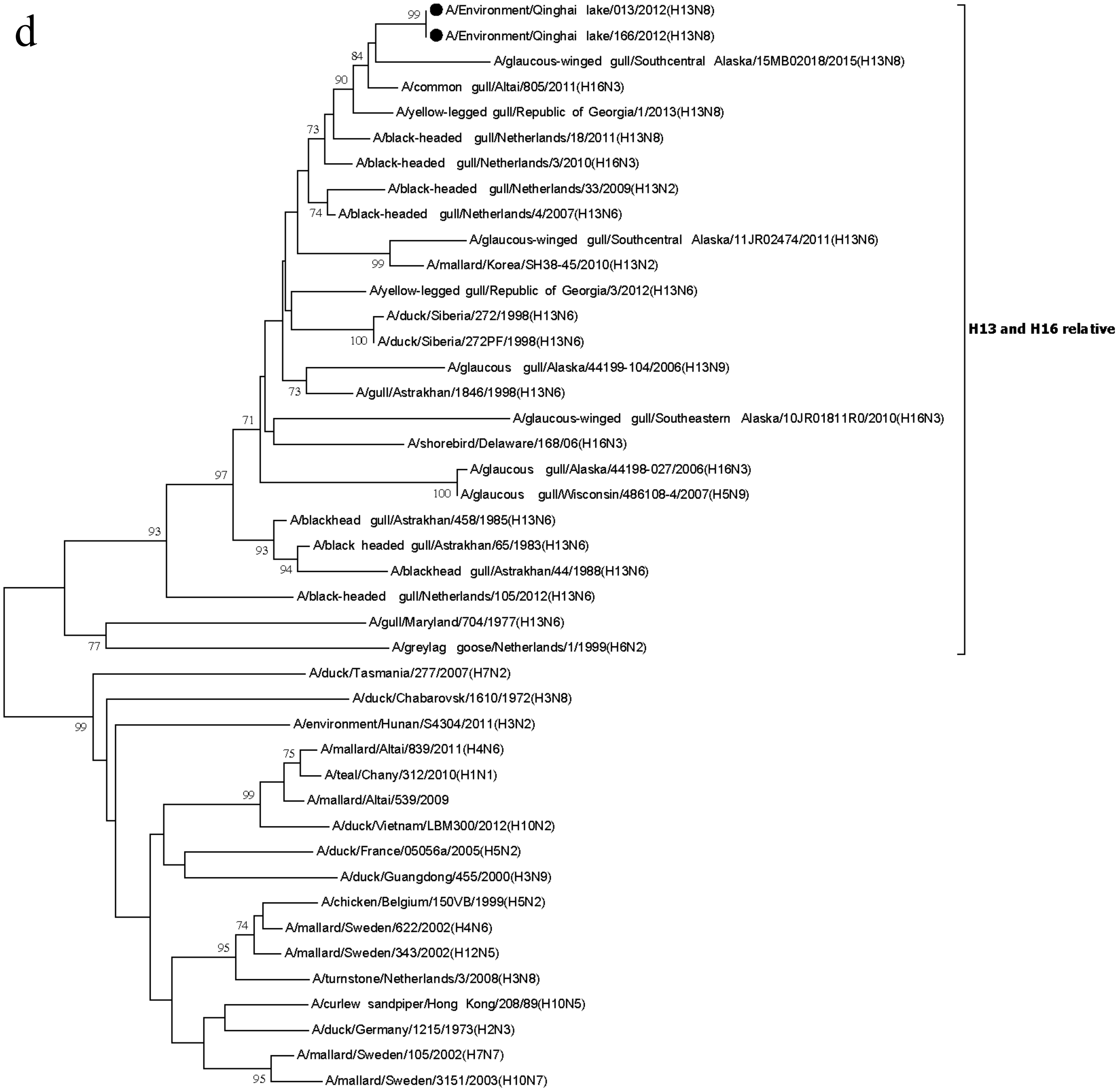

A/Environment/Qinghai lake/013/2012(H13N8)
A/Environment/Qinghai lake/166/2012(H13N8)
A/glaucous-winged gull/Southcentral Alaska/15MB02018/2015(H13N8)
A/common gull/Altai/805/2011(H16N3)
A/yellow-legged gull/Republic of Georgia/1/2013(H13N8)
A/black-headed gull/Netherlands/18/2011(H13N8)
A/black-headed gull/Netherlands/3/2010(H16N3)
A/black-headed gull/Netherlands/33/2009(H13N2)
A/black-headed gull/Netherlands/4/2007(H13N6)
A/glaucous-winged gull/Southcentral Alaska/11JR02474/2011(H13N6)
A/mallard/Korea/SH38-45/2010(H13N2)
A/yellow-legged gull/Republic of Georgia/3/2012(H13N6)
A/duck/Siberia/272/1998(H13N6)
A/duck/Siberia/272PF/1998(H13N6)
A/glaucous gull/Alaska/44199-104/2006(H13N9)
A/gull/Astrakhan/1846/1998(H13N6)
A/glaucous-winged gull/Southeastern Alaska/10JR01811R0/2010(H16N3)
A/shorebird/Delaware/168/06(H16N3)
A/glaucous gull/Alaska/44198-027/2006(H16N3)
A/glaucous gull/Wisconsin/486108-4/2007(H5N9)
A/blackhead gull/Astrakhan/458/1985(H13N6)
A/black headed gull/Astrakhan/65/1983(H13N6)
A/blackhead gull/Astrakhan/44/1988(H13N6)
A/black-headed gull/Netherlands/105/2012(H13N6)
A/gull/Maryland/704/1977(H13N6)
A/greylag goose/Netherlands/1/1999(H6N2)
A/duck/Tasmania/277/2007(H7N2)
A/duck/Chabarovsk/1610/1972(H3N8)
A/environment/Hunan/S4304/2011(H3N2)
A/mallard/Altai/839/2011(H4N6)
A/teal/Chany/312/2010(H1N1)
A/mallard/Altai/539/2009
A/duck/Vietnam/LBM300/2012(H10N2)
A/duck/France/05056a/2005(H5N2)
A/duck/Guangdong/455/2000(H3N9)
A/chicken/Belgium/150VB/1999(H5N2)
A/mallard/Sweden/622/2002(H4N6)
A/mallard/Sweden/343/2002(H12N5)
A/turnstone/Netherlands/3/2008(H3N8)
A/curlew sandpiper/Hong Kong/208/89(H10N5)
A/duck/Germany/1215/1973(H2N3)
A/mallard/Sweden/105/2002(H7N7)
A/mallard/Sweden/3151/2003(H10N7)
H13 and H16 relative
99 84 90 73 74 99 100 73 71 100 97 93 93 94 77 99 75 99 74 95 95

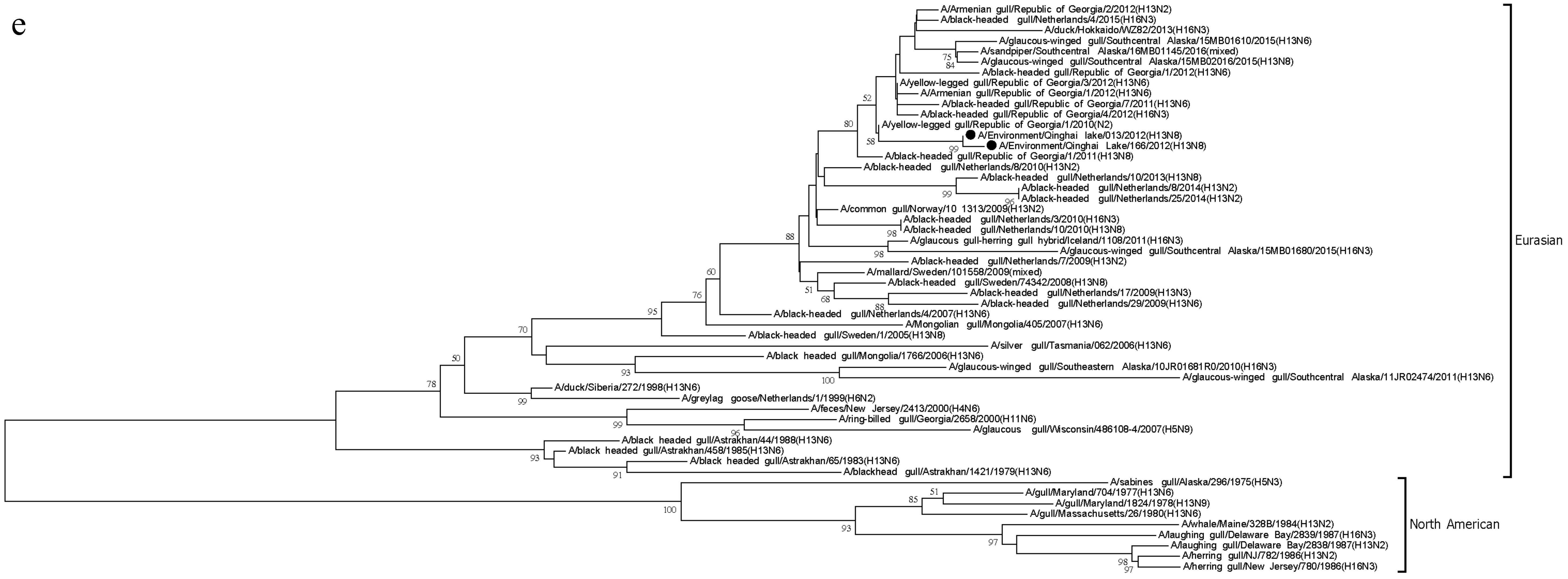
e
A/Armenian gull/Republic of Georgia/2/2012(H13N2)
A/black-headed gull/Netherlands/4/2015(H16N3)
A/duck/Hokkaido/WZ82/2013(H16N3)
A/glaucous-winged gull/Southcentral Alaska/15MB01610/2015(H13N6)
A/sandpiper/Southcentral Alaska/16MB01145/2016(mixed)
A/glaucous-winged gull/Southcentral Alaska/15MB02016/2015(H13N8)
A/black-headed gull/Republic of Georgia/1/2012(H13N6)
A/yellow-legged gull/Republic of Georgia/3/2012(H13N6)
A/Armenian gull/Republic of Georgia/1/2012(H13N6)
A/black-headed gull/Republic of Georgia/7/2011(H13N6)
A/black-headed gull/Republic of Georgia/4/2012(H16N3)
A/yellow-legged gull/Republic of Georgia/1/2010(N2)
A/Environment/Qinghai lake/013/2012(H13N8)
A/Environment/Qinghai Lake/166/2012(H13N8)
A/black-headed gull/Republic of Georgia/1/2011(H13N8)
A/black-headed gull/Netherlands/8/2010(H13N2)
A/black-headed gull/Netherlands/10/2013(H13N8)
A/black-headed gull/Netherlands/8/2014(H13N2)
A/black-headed gull/Netherlands/25/2014(H13N2)
A/common gull/Norway/10 1313/2009(H13N2)
A/black-headed gull/Netherlands/3/2010(H16N3)
A/black-headed gull/Netherlands/10/2010(H13N8)
A/glaucous gull-herring gull hybrid/Iceland/1108/2011(H16N3)
A/glaucous-winged gull/Southcentral Alaska/15MB01680/2015(H16N3)
A/black-headed gull/Netherlands/7/2009(H13N2)
A/mallard/Sweden/101558/2009(mixed)
A/black-headed gull/Sweden/74342/2008(H13N8)
A/black-headed gull/Netherlands/17/2009(H13N3)
A/black-headed gull/Netherlands/29/2009(H13N6)
A/black-headed gull/Netherlands/4/2007(H13N6)
A/Mongolian gull/Mongolia/405/2007(H13N6)
A/black-headed gull/Sweden/1/2005(H13N8)
A/silver gull/Tasmania/062/2006(H13N6)
A/black headed gull/Mongolia/1766/2006(H13N6)
A/glaucous-winged gull/Southeastern Alaska/10JR01681R0/2010(H16N3)
A/glaucous-winged gull/Southcentral Alaska/11JR02474/2011(H13N6)
A/duck/Siberia/272/1998(H13N6)
A/greylag goose/Netherlands/1/1999(H6N2)
A/feces/New Jersey/2413/2000(H4N6)
A/ring-billed gull/Georgia/2658/2000(H11N6)
A/glaucous gull/Wisconsin/486108-4/2007(H5N9)
A/black headed gull/Astrakhan/44/1988(H13N6)
A/black headed gull/Astrakhan/458/1985(H13N6)
A/black headed gull/Astrakhan/65/1983(H13N6)
A/blackhead gull/Astrakhan/1421/1979(H13N6)
A/sabines gull/Alaska/296/1975(H5N3)
A/gull/Maryland/704/1977(H13N6)
A/gull/Maryland/1824/1978(H13N9)
A/gull/Massachusetts/26/1980(H13N6)
A/whale/Maine/328B/1984(H13N2)
A/laughing gull/Delaware Bay/2839/1987(H16N3)
A/laughing gull/Delaware Bay/2838/1987(H13N2)
A/herring gull/NJ/782/1986(H13N2)
A/herring gull/New Jersey/780/1986(H16N3)
Eurasian
North American
75
84
52
80
58
99
99
95
98
98
88
51
68
88
60
76
95
70
50
78
93
100
99
99
96
93
91
100
51
85
93
97
98
97
